# Malignant transformation of struma ovarii into follicular thyroid carcinoma: A case report

**DOI:** 10.1097/MD.0000000000044240

**Published:** 2025-08-29

**Authors:** Yasushi Umezaki, Yasue Uriu, Kaoru Okugawa, Mariko Hashiguchi, Masatoshi Yokoyama

**Affiliations:** a Department of Obstetrics and Gynecology, Faculty of Medicine, Saga University, Saga, Japan; b Department of Pathology, Faculty of Medicine, Saga University, Saga, Japan.

**Keywords:** case report, follicular thyroid carcinoma, malignant transformation, mature cystic teratoma, ovarian struma ovarii

## Abstract

**Rationale:**

Struma ovarii is a rare form of mature cystic teratoma, with malignant transformation reported in approximately 5% to 10% of cases. Transformation into follicular thyroid carcinoma (FTC) is extremely uncommon; as a result, no standardized guidelines exist for treatment or prognosis for such cases.

**Patient concerns:**

A 54-year-old woman with cholelithiasis presented with upper abdominal discomfort. A lower abdominal mass was incidentally detected during evaluation.

**Diagnoses:**

Transvaginal ultrasonography revealed a 12-cm multilocular cystic tumor in the right ovary. Magnetic resonance imaging and computed tomography findings suggested a mature cystic teratoma with potential for malignant transformation. Exploratory laparotomy was performed, and intraoperative frozen section analysis confirmed malignant transformation.

**Interventions:**

The patient subsequently underwent total abdominal hysterectomy, bilateral salpingo-oophorectomy, pelvic lymphadenectomy, para-aortic lymph node biopsy, and omentectomy. Histopathological examination revealed tumor cells demonstrating cribriform proliferation, accompanied by eosinophilic structures, extensive necrosis, and venous invasion. These findings supported the diagnosis of FTC arising within struma ovarii.

**Outcomes:**

Postoperatively, the patient underwent routine follow-up, including serum thyroglobulin tests and imaging studies every 3 months. At 1 year, the patient showed no signs of tumor recurrence and remained in good clinical condition.

**Lessons:**

Malignant transformation of struma ovarii into FTC is an extremely rare condition and necessitates thorough histopathological evaluation for accurate diagnosis. This case highlights the diagnostic and therapeutic challenges associated with such tumors.

## 1. Introduction

Struma ovarii is a rare form of mature cystic teratoma, accounting for <1% of all ovarian tumors.^[1]^ Although most cases are benign, approximately 5% to 10% are reported to undergo malignant transformation.^[2]^ Among malignant cases, papillary thyroid carcinoma is the most commonly identified histological subtype, whereas follicular thyroid carcinoma (FTC) is extremely rare.^[3]^

FTC is characterized by an infiltrative growth pattern and strong potential for hematogenous metastasis, primarily affecting the lungs and bones.^[4]^ When FTC arises from struma ovarii, it may exhibit similar metastatic tendencies; however, its rarity limits clinical outcomes and optimal treatment strategies.

The mechanisms underlying the malignant transformation of struma ovarii remain unclear, with a recent study suggesting the role of genetic mutations, epigenetic alterations, and hormonal factors.^[5]^ Diagnosis is often challenging, requiring comprehensive histopathological evaluation and immunohistochemical analysis to distinguish FTC from other ovarian malignancies.^[6]^

Therefore, this report describes a rare case of malignant transformation of struma ovarii into FTC. The case highlights an extremely uncommon instance of FTC arising from struma ovarii. Given the diagnostic and therapeutic challenges posed by this condition, thorough histopathological evaluation and long-term follow-up are imperative. This case underscores the importance of maintaining a high index of suspicion in patients with complex ovarian masses and reinforces the need for individualized management strategies, which may guide other clinicians facing similar presentations.

Informed consent was obtained from the patient for the publication of this case and accompanying images. Sufficient measures were implemented to protect patient anonymity. This study was conducted with the approval of the Ethics Committee of Saga University Faculty of Medicine (2025-03-R-05). The authors declare no conflicts of interest relating to this study.

## 2. Case report

### 2.1. Patient information

A 54-year-old woman, gravida 4, para 2, was referred from another hospital and presented with epigastric discomfort. She had a past medical history of cholelithiasis. Her vital signs were stable, and no other significant past medical or family history was noted. Upon physical examination, a mobile mass was incidentally palpated in the lower abdominopelvic cavity.

### 2.2. Initial clinical findings

**Figure 1.**
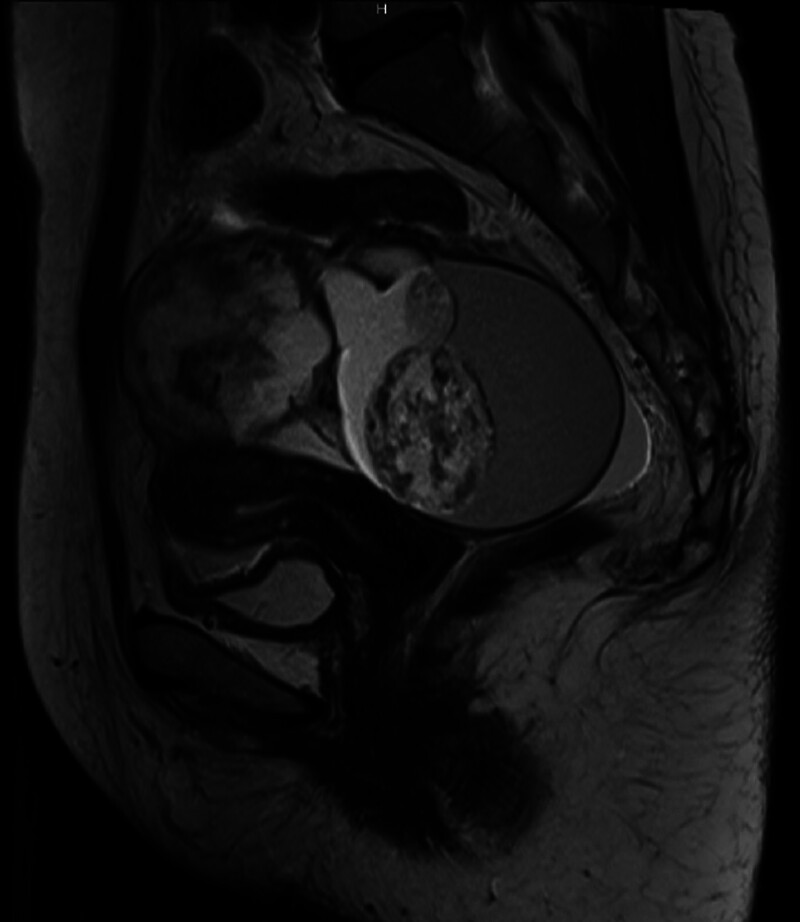
Sagittal T2-weighted MRI image. The enlarged cystic ovary contains solid components with high signal intensity.

Abdominal ultrasonography revealed the presence of gallstones. For further evaluation of a lower abdominal mass, transvaginal ultrasonography was performed and revealed a 12-cm multilocular cystic tumor in the right ovary, featuring thickened septa and solid components. A computed tomography scan demonstrated fat components and calcifications within the abdominal mass, suggesting a mature cystic teratoma of the right ovary. Magnetic resonance imaging confirmed the presence of solid areas and thickened septa, both enhanced with contrast (Fig. 1). Laboratory test results revealed elevated tumor markers, with cancer antigen 125 at 63 U/mL (reference, ≤35 U/mL) and cancer antigen 19-9 at 393 U/mL (reference, ≤37 U/mL), while carcinoembryonic antigen levels remained within normal limits.

### 2.3. Surgical procedure and initial histopathological diagnosis

As the epigastric discomfort was mild, cholelithiasis was scheduled for elective surgery at a later date and managed conservatively in the meantime. The lower abdominal mass, based on imaging findings and tumor marker profiles, raised suspicion of malignant transformation of a mature cystic teratoma, and an exploratory laparotomy was performed. Intraoperative frozen section analysis confirmed the presence of malignant features, prompting total abdominal hysterectomy, bilateral salpingo-oophorectomy, pelvic lymphadenectomy, para-aortic lymph node biopsy, and omentectomy.

### 2.4. Gross and histopathological findings

**Figure 2.**
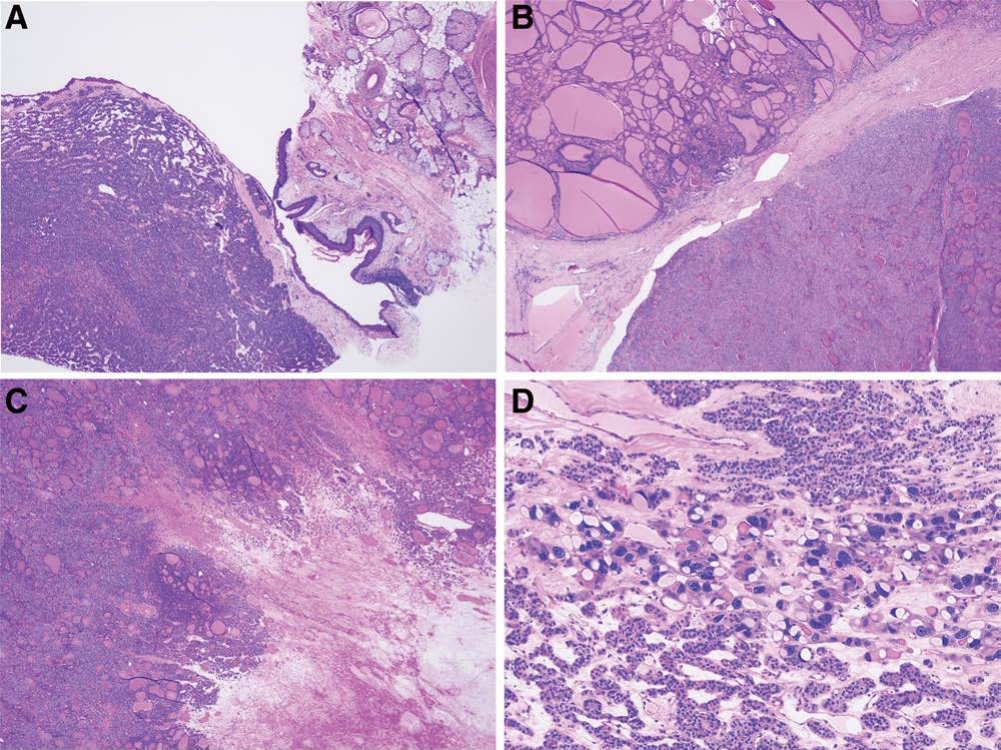
(A) Section showing the mature cystic teratoma (right) and follicular carcinoma components (left) (×20). (B) Section showing the struma ovarii (upper left) and follicular carcinoma component (lower left) (×20). (C) Necrotic area (lower left) surrounding the follicular carcinoma (×20). (D) Follicular carcinoma (center) (×100).

Gross pathological examination of the resected specimen revealed a cyst containing yellowish-white viscous fluid, hair, and sebaceous material. The cut surface demonstrated yellow, solid areas with clusters of small cysts. Microscopically, the tumor exhibited trabecular and small tubular growth patterns within an edematous stroma. The tubular lumina contained eosinophilic colloid-like material. Extensive necrosis and venous invasion were also observed (Fig. 2A–D). Immunohistochemistry demonstrated positive staining for thyroglobulin and thyroid transcription factor-1, confirming the thyroidal origin of the tumor. A final diagnosis of FTC arising from struma ovarii, with malignant transformation of a mature cystic teratoma, was made based solely on histopathological assessment.

### 2.5. Postoperative course and follow-up

Although postoperative adjuvant therapy was considered, no distant metastases were identified, and the malignant findings were confined to the ovary, thus supporting a decision to proceed with close monitoring. The patient was placed on a regular follow-up regimen, including serum thyroglobulin tests and imaging studies every 3 months. At 12 months postoperatively, no signs of recurrence were observed, and the patient remained in good clinical condition.

## 3. Discussion

The case represents an extremely rare occurrence of malignant transformation of struma ovarii into FTC. Accurate diagnosis relied on thorough histopathological evaluation, and the determination of optimal treatment strategies was required.

### 3.1. Histopathological characteristics

Malignant transformation of a mature cystic teratoma is diagnosed based on histopathological features, including increased cellular atypia, elevated cell density, vascular invasion, and capsular infiltration.^[1]^ In the present case, the tumor displayed cribriform growth patterns and eosinophilic colloid-like material within the luminal spaces. Extensive necrosis and venous invasion were also observed, strongly suggesting FTC.

Immunohistochemical analysis showed positive staining for thyroglobulin and thyroid transcription factor-1, confirming the thyroidal origin of the tumor. These findings were critical for differentiating ovarian FTC from other ovarian malignancies, particularly high-grade serous carcinoma and endometrioid carcinoma.^[2]^

FTC is characterized by an infiltrative growth pattern and a high potential for hematogenous metastasis.^[3]^ FTC arising from struma ovarii may exhibit similar metastatic tendencies; however, clinical data remain insufficient due to the rarity of such cases.

### 3.2. Treatment strategies and prognosis

The patient in this case did not present with distant metastases, and the malignant findings were confined to the ovary. Consequently, postoperative adjuvant therapy was withheld, and the patient was monitored closely instead.

Standard treatment for FTC of the thyroid typically involves total thyroidectomy followed by radioactive iodine therapy. However, managing FTC arising from struma ovarii remains challenging due to the rarity of the condition. Current management strategies are informed by a limited number of case reports and expert opinions.^[7]^ For tumors confined to the ovary, complete cytoreductive surgery is the primary treatment approach. In cases with distant metastases, radioactive iodine therapy may be considered if the tumor retains iodine uptake capability.^[8]^ However, the efficacy of radioactive iodine therapy in FTC arising from struma ovarii remains uncertain, as there is limited data on its responsiveness in this specific context.^[9]^

Postoperative monitoring is crucial for patient management. Serum thyroglobulin levels should be regularly measured, as t elevated levels may indicate recurrence or metastatic progression.^[10]^ Compared to papillary thyroid carcinoma, FTC exhibits a higher recurrence rate and worse prognosis, particularly in the presence of undifferentiated components.^[11]^ Thus, long-term follow-up is essential to detect recurrence early and manage metastases effectively.

For patients with radioactive iodine-refractory disease, tyrosine kinase inhibitors, including sorafenib and lenvatinib, have shown potential in clinical trials.^[12]^ However, the role of aggressive therapy in malignant struma ovarii remains debated. Recent reports suggest that a more conservative approach may be appropriate in the absence of extra-ovarian spread.^[13]^ Further studies are required to establish standardized treatment protocols for FTC arising from struma ovarii.

## 4. Conclusion

This case highlights an extremely rare occurrence of FTC arising from struma ovarii. Given the diagnostic and therapeutic challenges presented by this condition, thorough histopathological evaluation and long-term follow-up are essential. Future research in genetic and molecular profiling is crucial to elucidate malignant transformation mechanisms and optimize treatment strategies.

## Author contributions

**Conceptualization:** Yasushi Umezaki.

**Data curation:** Yasue Uriu.

**Investigation:** Mariko Hashiguchi.

**Supervision:** Kaoru Okugawa, Masatoshi Yokoyama.

**Writing – review & editing:** Yasushi Umezaki.
